# Stratified Analysis of Patients Within the PSA Gray Zone (4–10 ng/mL) and Its Clinical Application Value: Development of a Predictive Model for Clinically Significant Prostate Cancer Using Quantitative Indicators of PSA, ADC, and Relative T2 Value

**DOI:** 10.3390/diagnostics16172771

**Published:** 2026-08-28

**Authors:** Pan Hao, Tong Zhu, Ruiqiang Xin, Xiaoyong Lv

**Affiliations:** Medical Imaging Center, LuHe Hospital, Capital Medical University, Beijing 101100, China; xiaopan217511@163.com (P.H.); zhutong@mail.ccmu.edu.cn (T.Z.); lvxiaoyonging@163.com (X.L.)

**Keywords:** clinically significant prostate cancer, PSA gray zone, relative T2 value, nomogram, risk stratification

## Abstract

**Objective**: Patients with prostate-specific antigen (PSA) levels in the 4–10 ng/mL gray zone present a diagnostic challenge, as PSA alone cannot reliably distinguish clinically significant prostate cancer (csPCa) from benign conditions or indolent disease, potentially leading to unnecessary biopsies or missed clinically significant disease. This study aimed to develop a risk prediction model for csPCa in this population using clinical indicators and magnetic resonance imaging (MRI) quantitative data, and to evaluate its potential value for risk stratification and as a supplementary tool for biopsy decision-making. **Methods**: We retrospectively included 210 patients with PSA levels in the 4–10 ng/mL range and confirmed pathological diagnoses, who were admitted to our hospital between January 2018 and June 2025. csPCa was defined as a Gleason score ≥ 7. Patients were stratified by pathological diagnosis (csPCa vs. non-csPCa) and randomly divided into training and internal validation sets in a 7:3 ratio. The following variables were collected for analysis: age, PSA, apparent diffusion coefficient (ADC), and relative T2 value. Univariate and multivariate logistic regression were used to identify independent predictors of csPCa. We constructed a prediction model and nomogram. The discriminating ability, calibration, and stability of the model were assessed using receiver operating characteristic (ROC) curves, calibration curves, and Bootstrap internal validation. Decision curve analysis (DCA) was performed to evaluate the model’s net benefit across clinically relevant threshold probabilities. Risk stratification was performed based on predicted probabilities. **Results**: Of the 210 patients, 50 had csPCa and 160 had non-csPCa lesions. Univariate regression showed associations between age, ADC value, and relative T2 value. Multivariate logistic regression identified age and relative T2 value as independent predictors in the final model. Internal validation using 1000 Bootstrap resampling with optimism correction yielded a corrected AUC of 0.698 (95% CI: 0.669–0.716), with a mean optimism of only 0.016, indicating minimal overfitting. The area under the curve (AUC) of the model was 0.720 in the training set and 0.711 in the validation set. The calibration curve for the training set demonstrated good agreement between predicted and observed probabilities. DCA showed net benefits across certain threshold probabilities. Risk stratification based on predicted probability showed csPCa detection rates of 14.7%, 28.3%, and 47.4% in low-, intermediate- and high-risk groups, respectively. **Conclusions**: The logistic binary classification model based on age and relative T2 value may assist in risk assessment for csPCa in PSA gray zone patients. However, its moderate discrimination suggests it should be used as a supplementary tool rather than a standalone diagnostic test. Risk stratification based on predicted probability may help identify different csPCa risk populations, but given the exploratory nature of this single-center retrospective study, multi-center, large-sample, and prospective studies are required for external validation and optimization before clinical implementation.

## 1. Introduction

Prostate-specific antigen (PSA) is the best-known serological biomarker for prostate cancer (PCa) detection. It is used for clinical screening and disease monitoring, and plays a key role in early detection of PCa [1]. PSA is not exclusively associated with PCa, as non-malignant conditions such as benign prostatic hyperplasia and prostatitis can also elevate PSA levels. The PSA range between 4 and 10 ng/mL (called the PSA gray zone) is challenging to diagnose. Within this range, the PCa detection rate is only 15% to 25%, and over 75% of cases are benign prostatic hyperplasia or prostatitis. The overlap between benign and malignant features complicates the decision to perform a biopsy based solely on PSA levels, making it difficult to determine the presence of PCa and often leading to unnecessary procedures or missed diagnoses [2]. Age is a major risk factor for PCa; the incidence increases with advancing age. Previous studies have shown that age is linked to PCa but also plays an important role in risk stratification and predictive models [3,4,5,6].

The difficulty in diagnosing patients in the PSA gray zone arises in three ways. First, the uncertainty of disease probability makes clinical decisions difficult, given that there is not enough malignant evidence for a biopsy and enough benign evidence to dismiss it. There is also the double risk of missed diagnoses and unnecessary biopsies. Performing biopsies on all patients in this PSA range results in over 75% unnecessary procedures, leading to bleeding, infections, and urinary incontinence. Second, conservative observation could mean missing about 20% of clinically significant prostate cancer (csPCa) cases, delaying treatment, and affecting patient outcomes [7]. Third, current diagnostic methods do not fully meet clinical needs. The Prostate Imaging Reporting and Data System (PI-RADS) relies on subjective interpretation, which can lead to inconsistent diagnoses. Digital rectal examination has low specificity, and magnetic resonance imaging (MRI) quantitative indicators such as the apparent diffusion coefficient (ADC) or absolute T2 signal intensity are used for general prostate lesion diagnoses, although their effectiveness is limited when used in isolation. Overall, there remains a need for objective and reproducible quantitative tools that can supplement existing clinical assessment and assist in risk stratification [8].

Several previous studies have investigated risk prediction models for PCa in the PSA gray zone. Polihronidou et al. systematically reviewed predictive factors and identified age, PSA density, and MRI findings as key predictors [3]. Guo et al. conducted a meta-analysis demonstrating that MRI has moderate diagnostic performance in this population [2]. Chai et al. constructed a nomogram for biopsy-naïve patients with PSA < 10 ng/mL and PI-RADS ≤ 3 lesions [9], while Wu and Tang highlighted the complementary value of combining PSA density with multiparametric MRI [10].

Despite these advances, several gaps remain in the existing literature. First, most previous models incorporated subjective imaging parameters such as PI-RADS scores, which are prone to inter-observer variability and may limit model reproducibility. Second, while PSA density and f/t PSA ratio have been widely studied, these variables are not consistently available in routine clinical practice, particularly in primary care or retrospectively collected cohorts. Third, the measurement of quantitative MRI parameters such as ADC and absolute T2 signal intensity lacks standardization across studies, making direct comparisons difficult. Fourth, few studies have specifically addressed the PSA gray zone population with a parsimonious model based on readily available and objectively quantifiable indicators. Fifth, most existing models have not provided practical risk stratification strategies with clear clinical action thresholds for biopsy decision-making in this population.

To address these gaps, this study focused specifically on csPCa, defined as Gleason score ≥ 7 (ISUP Grade Group ≥ 2), and made the following contributions. First, we introduced the relative T2 value as a standardized MRI indicator, normalizing lesion signal to normal tissue at the same level to reduce inter-scanner and inter-patient variability. Second, we developed a parsimonious binary classification model using only age and relative T2 value—both objectively quantifiable and routinely available—avoiding subjective scoring or inconsistently recorded laboratory variables. Third, we established a three-tier risk stratification system with clearly defined clinical action thresholds. Fourth, we visualized the model as a nomogram for bedside use. Fifth, we performed rigorous Bootstrap internal validation with optimism correction to provide a reliable performance estimate for csPCa prediction despite the limited sample size.

In this study, we combined clinical data with standard MRI quantitative indicators to identify the predictors of csPCa and develop a visual risk prediction model for the PSA gray zone population. We assessed the discriminative ability, calibration, and net benefit using split-sample validation, Bootstrap correction, calibration curve, and decision curve analysis (DCA), with the aim of providing an objective and supplementary tool for individualized csPCa risk assessment and risk stratification.

## 2. Materials and Methods

### 2.1. Patient Selection

This retrospective study involved 210 patients with serum PSA levels between 4 and 10 ng/mL evaluated at our hospital from January 2018 to June 2025. All patients were tested for serum PSA and prostate MRI before pathological confirmation. Pathological diagnosis was obtained via transrectal ultrasound-guided biopsy or radical prostatectomy. Prostate biopsy specimens served as the reference standard in 198 patients (94.3%), whereas radical prostatectomy specimens served as the reference standard in 12 patients (5.7%). csPCa was defined as a Gleason score ≥ 7 (ISUP Grade Group ≥ 2). When both were available, the radical prostatectomy specimen was used as the reference standard. The Medical Ethics Committee of our hospital reviewed and approved the study protocol. All patient data were anonymized, and informed consent was waived. All research procedures were conducted strictly according to the Declaration of Helsinki.

Inclusion criteria: (1) age 50–75 years; (2) PSA levels in the 4–10 ng/mL range, with MRI performed within 2 weeks of the PSA test; (3) clinical suspicion of prostate disease (e.g., prostate nodules on digital rectal examination or abnormal urinalysis), warranting MRI and pathological biopsy; (4) no prior targeted therapy, surgery, radiotherapy, or hormonal therapy before biopsy.

Exclusion criteria: (1) contraindications to MRI or poor MRI image quality (e.g., motion artifacts, unclear lesion visualization, or inadequate quality for quantitative measurement); (2) presence of other malignant tumors in the urinary tract; (3) lack of clinical, pathological, or quantitative imaging data.

### 2.2. Observation Indicators and Quantitative Standards

Pathological findings were used as the reference standard for classification of csPCa and non-csPCa. The following variables were collected for analysis: clinical indicators, serological markers, and quantitative MRI parameters. MRI examinations were performed using a 3.0-T uMR 780 MRI system (Shanghai United Imaging, Shanghai, China) with built-in coils. The imaging protocol included axial T1-weighted imaging (T1WI), T2-weighted imaging (T2WI), and diffusion-weighted imaging (DWI). For T1WI, the acquisition parameters were as follows: TR/TE = 640/11 ms, slice thickness = 4 mm, inter-slice gap = 1 mm, matrix = 320 × 224, field of view (FOV) = 28 × 28 cm, and number of excitations (NEX) = 2. For T2WI, the parameters were TR/TE = 3600/100 ms, slice thickness = 4 mm, inter-slice gap = 1 mm, matrix = 320 × 224, FOV = 28 × 28 cm, and NEX = 4. For DWI, the parameters were TR/TE = 5000/90 ms, slice thickness = 4 mm, inter-slice gap = 1 mm, matrix = 96 × 128, FOV = 28 × 28 cm, and NEX = 4. Each indicator was standardized and measured quantitatively as follows. Serum total PSA levels were measured using chemiluminescence immunoassay with a corresponding commercial immunoassay reagent kit. PSA testing was performed within 2 weeks before or after the MRI examination, and values were recorded in ng/mL. ADC values were derived from the diffusion-weighted imaging (DWI) sequence (b = 0 and 1500 s/mm^2^), with ADC maps generated using syngo.via (version VB60; Siemens, Munich, Germany) on a syngo Workplace workstation. A circular or oval region of interest (ROI) was delineated at the level where the lesion demonstrated its maximum cross-sectional area, covering approximately 50–66% of the lesion area, while avoiding the urethra, blood vessels, calcifications, cysts, and other non-lesion structures. Measurements were repeated twice at the same location, and the mean value was used for analysis and expressed as ×10^−3^ mm^2^/s.

Relative T2 value: On T2-weighted imaging (T2WI), an ROI was placed on the lesion at the same level, with the same size and location as that used for ADC measurement, to quantify the T2 signal intensity. Another ROI of identical size was placed in the normal-appearing peripheral zone at the same level. The relative T2 value was then calculated as the ratio of the T2 signal intensity of the lesion to that of the normal peripheral zone tissue.

### 2.3. Data Collection and Quality Control

Data collection: Two researchers (both trained on standard training) searched the hospital electronic medical record system and picture archive and communication system (PACS). They extracted clinical data (age, PSA values), imaging data (MRI original images, DWI/ADC reconstructed images), and pathology data (pathological diagnosis results) from PACS. A single database was created and two people checked data entries. Two radiologists (each with >5 years of experience in prostate MRI interpretation, attending physician or higher) independently measured ADC and T2 signal intensities using standardized ROI placement, blinded to each other’s results. Interobserver agreement was evaluated with the ICC. If the ICC was <0.75, measurements were repeated after discussion; if ≥0.75, the mean values were used [11]. Pathological diagnosis was based on tissue obtained via transrectal ultrasound-guided biopsy or radical prostatectomy. When both methods were available, the radical prostatectomy specimen was used as the reference standard.

### 2.4. Statistical Methods

We used MedCalc 23.0 and R version 4.2.3 (R Foundation for Statistical Computing, Vienna, Austria), including the rms, pROC, and dca packages for statistical analysis and graphing. All statistical tests were two-sided, and a *p* value of <0.05 was considered statistically significant.

#### 2.4.1. Statistical Analyses and Comparisons

The Shapiro–Wilk test was used to assess normality of continuous variables. Normally distributed variables were expressed as mean ± standard deviation (SD) and compared between groups using the independent-samples *t*-test. Non-normally distributed variables were expressed as median (interquartile range, IQR) and compared using the Mann–Whitney U test. Categorical variables were presented as frequencies (percentages) and compared using the chi-square test. All patients were classified into csPCa and non-csPCa groups based on pathological results.

#### 2.4.2. Dataset Partitioning and Preprocessing

The subjects were stratified by pathological diagnosis (csPCa/non-csPCa) and then randomly divided into training and internal validation sets in a 7:3 ratio. To prevent data leakage, all patient data were partitioned strictly at the individual patient level, ensuring that data from the same patient were not simultaneously assigned to both sets. All data preprocessing steps—including any potential normalization or variable transformation—were performed exclusively within the training set. The derived preprocessing parameters were then applied to the internal validation set. Given the relatively limited sample size of the PSA gray zone cohort (*n* = 210) and the limited number of csPCa events, further division into training, validation, and independent test sets would have reduced the statistical power for model development. Therefore, bootstrap resampling with 1000 iterations was additionally performed for internal validation and optimism correction. The 7:3 split-sample analysis was used as a secondary internal validation approach. External validation in independent multicenter cohorts across different MRI platforms and patient populations remains necessary to assess model generalizability.

#### 2.4.3. Variable Screening and Classification Model Construction

Univariate analysis was performed on the training set to identify candidate predictors, which were then entered into a multivariate logistic regression model to identify independent predictors. A binary logistic regression classification model (csPCa = 1, non-csPCa = 0) was developed to predict the individual probability of csPCa.

#### 2.4.4. Risk Stratification Calculation

Using the final logistic regression prediction model, we calculated the predicted probability of csPCa for each patient. The optimal cutoff value was initially determined by maximizing the Youden index from the receiver operating characteristic (ROC) curve in the training set, yielding a threshold of approximately 0.30. To facilitate clinically interpretable risk grouping, we further established buffer zones around this cutoff to create a three-tier risk stratification system. Specifically, patients with predicted probability *p* < 0.20 were classified as low-risk, those with 0.20 ≤ *p* < 0.40 as intermediate-risk, and those with *p* ≥ 0.40 as high-risk. These thresholds were used for exploratory risk stratification rather than as definitive biopsy decision thresholds, with the aim of distinguishing patients with different estimated risks of csPCa.

#### 2.4.5. Model Performance Validation and Evaluation

The primary method for internal validation was Bootstrap resampling with 1000 iterations, which provides a more robust estimate of model performance than a single train–test split. The 7:3 split-sample validation was performed as a secondary check. Model performance was assessed in terms of discrimination, calibration, internal stability, and potential decision-analytic value. The discriminative performance was evaluated by plotting ROC curves and calculating the area under the curve (AUC) with 95% confidence intervals (95% CIs), together with sensitivity, specificity, and Youden index. To formally evaluate the incremental predictive value of adding relative T2 value to age, we compared the full model (age + relative T2 value) with a baseline model containing age alone using the likelihood ratio test and continuous net reclassification improvement (NRI) with 95% CIs. According to the criteria proposed by Mandrekar [12], an AUC > 0.9 was considered excellent, 0.8–0.9 good, 0.7–0.8 fair, and <0.7 poor [13]. Model calibration was assessed in both the training and internal validation sets using the Hosmer–Lemeshow goodness-of-fit test, calibration curves, and the Brier score. Calibration slope and intercept were additionally estimated to quantify calibration performance. DCA was performed to compare the net benefit of the model with the “biopsy all” and “biopsy none” strategies across a range of threshold probabilities [14]. Numerical net benefits at threshold probabilities of 0.20, 0.30, and 0.40 were additionally calculated. As a sensitivity analysis, ADC was forced into the final model to construct an extended model (age + relative T2 value + ADC), which was compared with the primary model (age + relative T2 value) to assess whether ADC provided incremental predictive value.

#### 2.4.6. Consistency Inspection

Interobserver agreement for ADC and relative T2 measurements was assessed using the intraclass correlation coefficient (ICC). An ICC > 0.90 was considered excellent, 0.75–0.90 good, 0.50–0.75 moderate, and <0.50 poor [11].

## 3. Results

### 3.1. Baseline Characteristics and Interobserver Agreement

**Figure 1:**
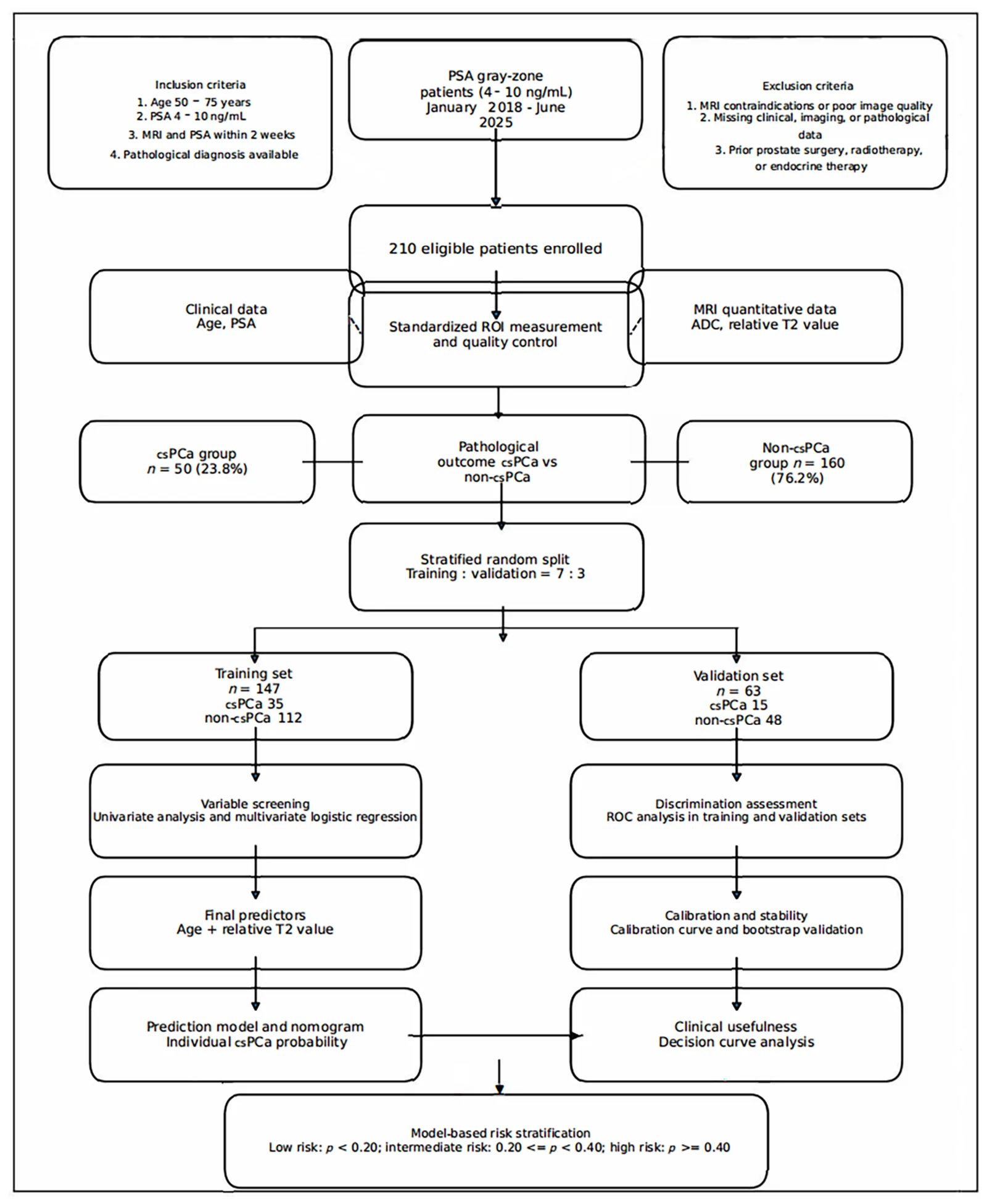
Flowchart of patient inclusion and grouping in this study.

The enrollment and grouping process of the research subjects is shown in Figure 1.

This study included 210 patients in the PSA gray zone (4–10 ng/mL) with 50 (23.81%) csPCa cases and 160 (76.19%) non-csPCa cases. The median age of the csPCa group was 65.5 years (range 62.0–70.0), significantly higher than the age of the non-csPCa group (range 61.0–67.0, *p* = 0.034). The non-csPCa group had higher ADC values (1.17 ± 0.17 vs. 1.09 ± 0.15 × 10^−3^ mm^2^/s, *p* < 0.001) and lower relative T2 values (1.10 ± 0.17 vs. 1.20 ± 0.15, *p* < 0.001) than the csPCa group. PSA levels were not significantly different between the groups (*p* = 0.718) (Table 1). Interobserver agreement for ADC and relative T2 measurements was excellent, with ICC values exceeding 0.9 for both single and average measures (Table 2).

A total of 210 patients in the PSA gray zone were stratified by pathological diagnosis (csPCa/non-csPCa) and randomly divided into training and internal validation sets in a 7:3 ratio. The training set consisted of 147 cases (70.00%), including 35 cases (23.81%) of csPCa and 112 cases (76.19%) of non-csPCa. The internal validation set consisted of 63 cases (30.00%), including 15 cases (23.81%) of csPCa and 48 cases (76.19%) of non-csPCa.

Training and internal validation sets were comparable in age (64.0 [62.0–67.0] vs. 64.0 [61.3–68.0] years, Z = −0.147, *p* = 0.445), PSA (6.73 [6.01–7.42] vs. 6.76 [6.18–7.46] ng/mL, Z = 0.211, *p* = 0.833), ADC (1.10 ± 0.15 vs. 1.12 ± 0.17 ×10^−3^ mm^2^/s, t = 0.905, *p* = 0.366) and relative T2 value (1.12 ± 0.17 vs. 1.13 ± 0.18, t = 0.423, *p* = 0.673). There were no statistically significant differences in the distribution of csPCa and non-csPCa between the training and internal validation sets (23.8%/76.2% vs. 23.8%/76.2%, χ^2^ = 0, *p* = 1.000) (Table 3).

### 3.2. Model Construction and Diagnostic Efficacy Evaluation

#### 3.2.1. Screening of Core Predictors for the Model

**Figure 2:**
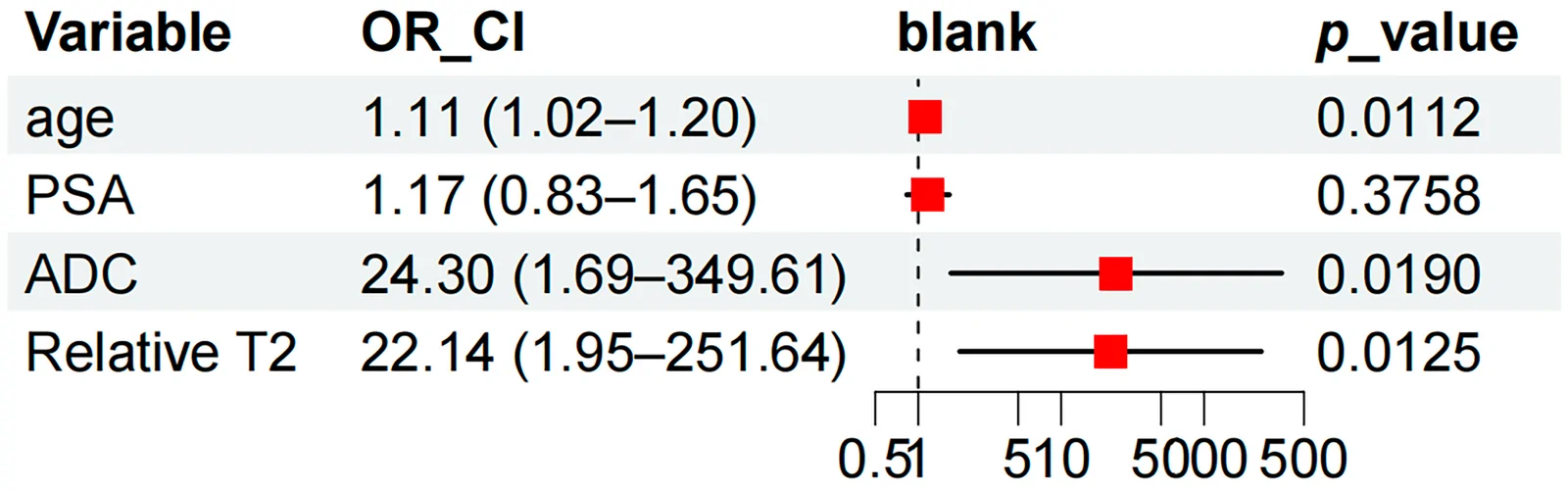
Univariate logistic regression analysis of predictors for csPCa.

Univariate analysis was performed for each variable, and showed statistically significant differences in age, ADC value and relative T2 value between csPCa and non-csPCa groups (all *p* < 0.05) (Figure 2). These significant variables were then included in a multivariate logistic regression analysis. Age (OR = 1.11, 95% CI = 1.02–1.20, *p* = 0.017) and relative T2 value (OR = 22.40, 95% CI = 1.88–267.19, *p* = 0.014) were independent predictors of csPCa. With 50 csPCa events and 2 predictors in the final model, the events per variable (EPV) was 25, which exceeds the recommended minimum of 10 for logistic regression analysis. However, the wide confidence interval for the relative T2 value indicated limited precision of the effect estimate. Age and relative T2 value were used to construct the primary prediction model (Table 4).

#### 3.2.2. Construction of Logistic Binary Classification Prediction Model and Nomogram

**Figure 3:**
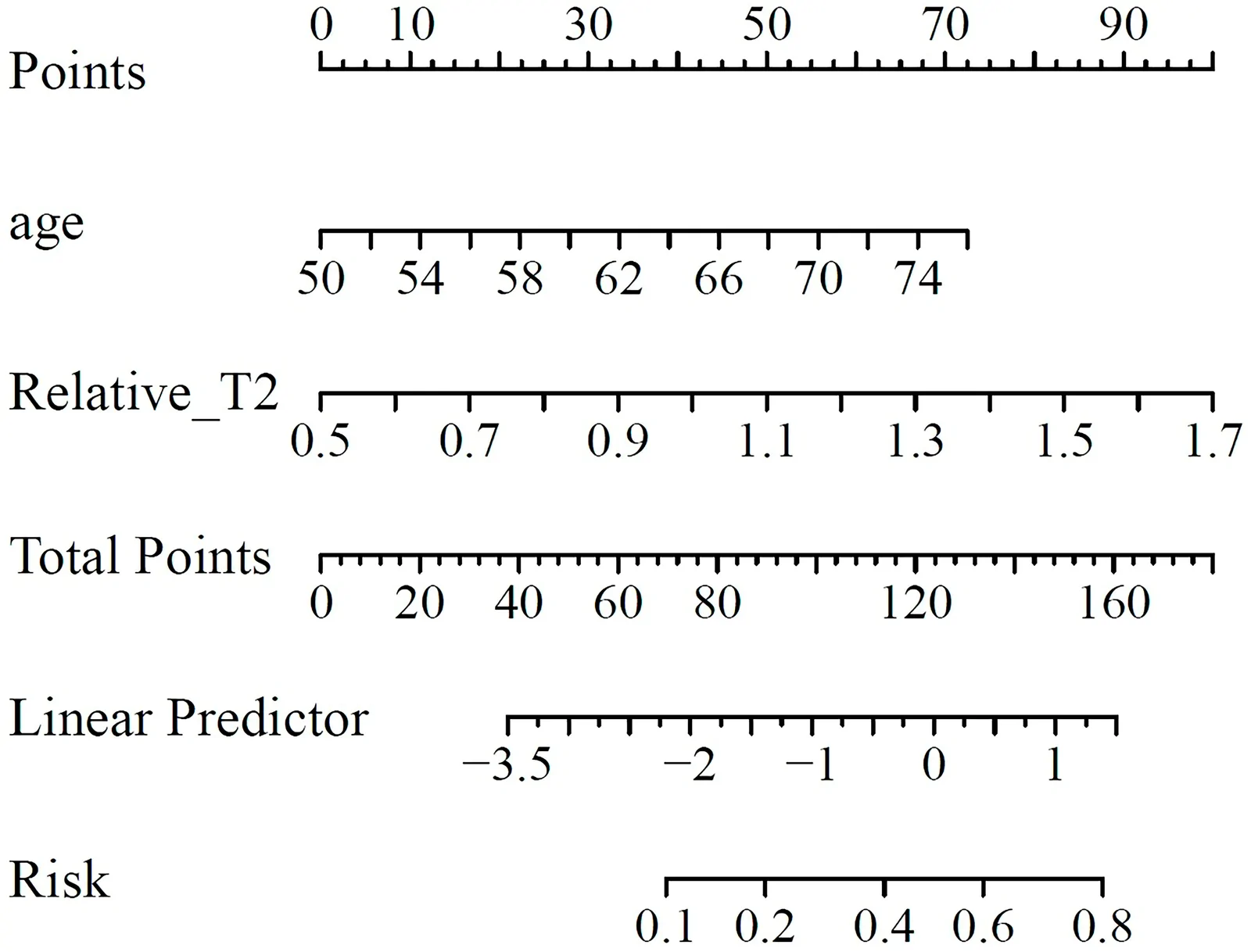
A nomogram for predicting the risk of csPCa using age and relative T2 value as key variables.

A binary logistic regression prediction model for csPCa was developed using age and relative T2 value as independent predictors, with pathological diagnosis as the dependent variable. A nomogram was constructed to visualize the model for individual risk assessment (Figure 3).

#### 3.2.3. Evaluation of Model Interpretability and Diagnostic Performance

**Figure 4:**
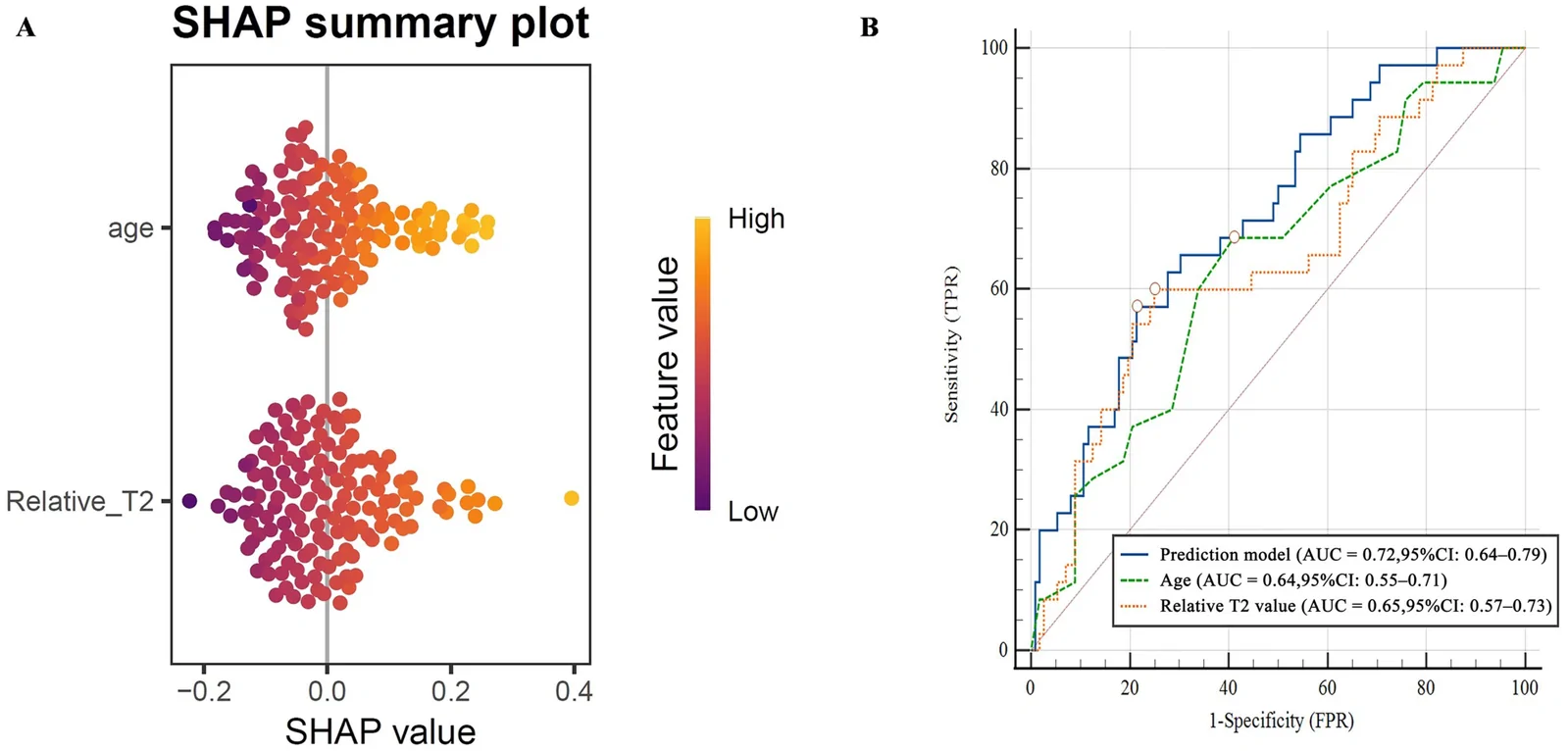
Interpretability and diagnostic performance of the prediction model for csPCa. (**A**): The SHAP summary plot illustrates the contributions of age and relative T2 value to the prediction model. (**B**): ROC curves compare the prediction model, age, and relative T2 value in differentiating csPCa from non-csPCa.

SHAP (SHapley Additive exPlanations) analysis was used to assess each variable’s contribution to model predictions. As shown in Figure 4A, both age and relative T2 value were associated with higher risk of PCa. The combined model demonstrated superior diagnostic performance compared with either variable alone, achieving an AUC of 0.72 (95% CI: 0.64–0.79), versus 0.64 (95% CI: 0.55–0.71) for age alone and 0.65 (95% CI: 0.57–0.73) for relative T2 value alone (Figure 4B).

#### 3.2.4. Internal Validation of Model Diagnostic Efficacy

**Figure 5:**
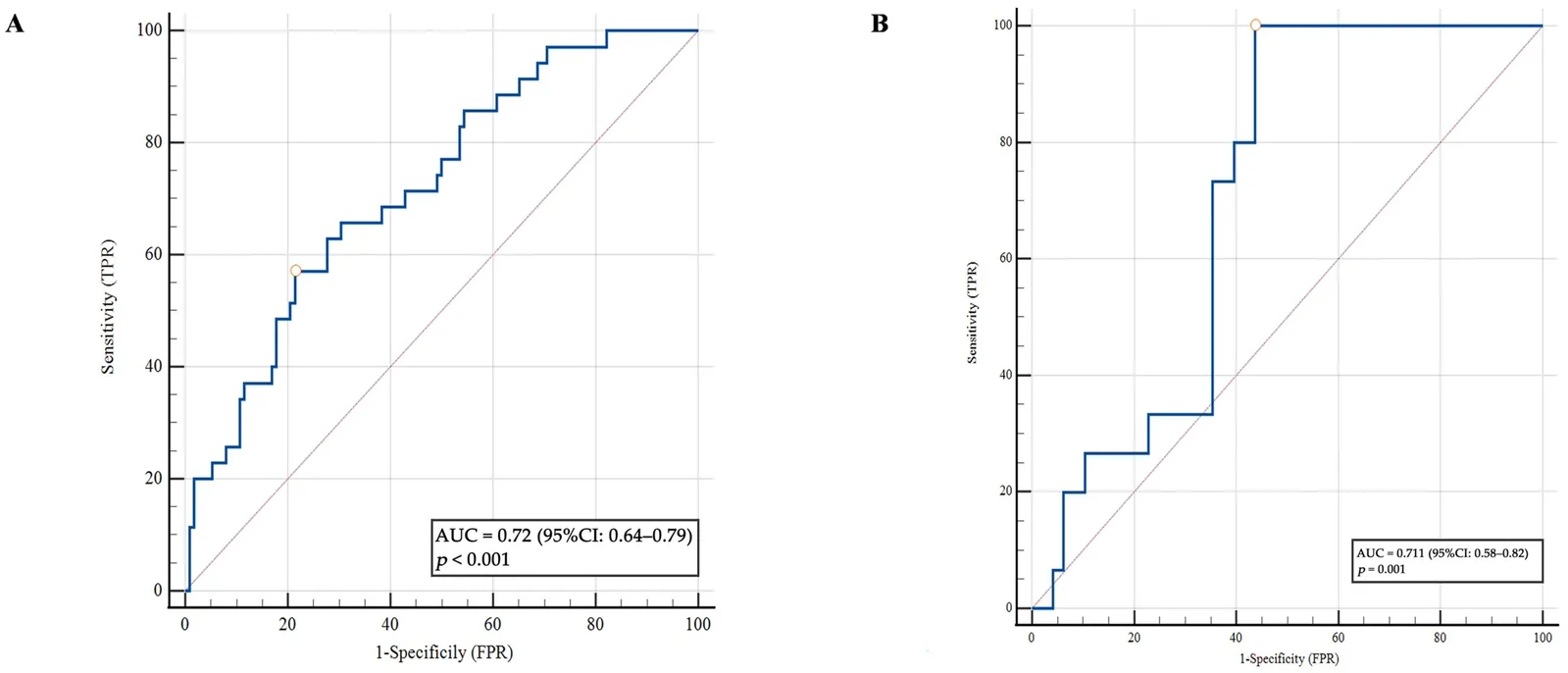
ROC curves of the prediction model for csPCa in the training and internal validation sets. (**A**): Training set. (**B**): Internal validation set.

In the training set, the predictive model combining age and relative T2 value achieved an AUC of 0.720 (95% CI: 0.64–0.79, *p* < 0.001) for distinguishing csPCa from non-csPCa. In the internal validation set, the AUC was 0.711 (95% CI: 0.58–0.82, *p* < 0.001), which was similar to that in the training set. These findings suggest that the model maintained moderate discriminative performance in the internal validation set (Figure 5). The likelihood ratio test showed that the full model significantly improved model fit compared with the baseline model (χ^2^ = 7.84, *p* = 0.005). The continuous NRI was 0.105 (95% CI: −0.105 to 0.315). The point estimate suggests some improvement in risk classification with the addition of relative T2 value to age, although the wide confidence interval indicates that this finding requires further validation in larger cohorts. This is consistent with the observed AUC improvement from 0.64 for age alone to 0.72 for the full model, suggesting potential incremental predictive value of relative T2 value beyond age.

#### 3.2.5. Model Calibration Evaluation

**Figure 6:**
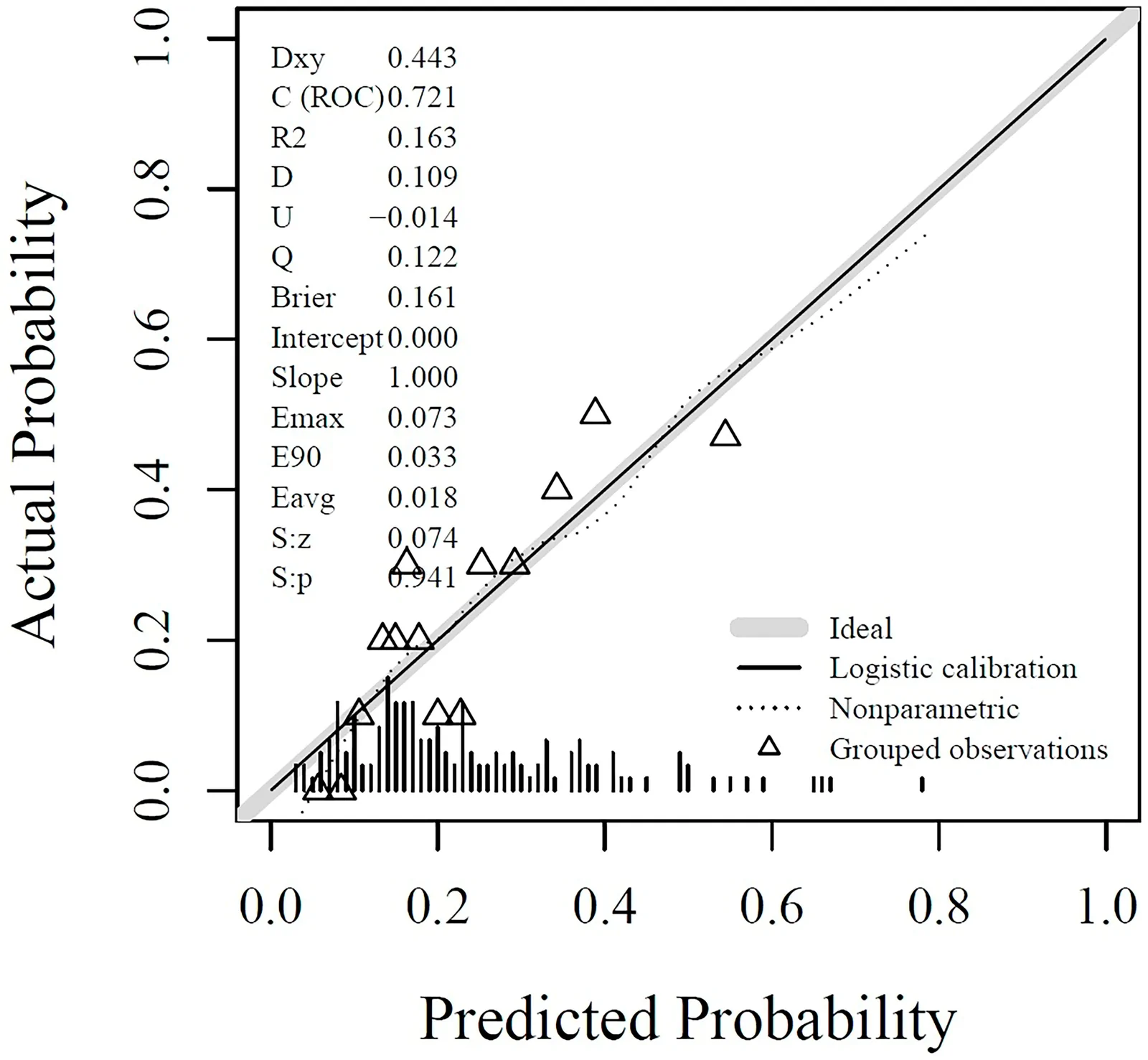
Calibration curve of the prediction model in the training set.

The calibration curve showed that the model’s prediction probabilities were close to observed probabilities. With a C-index of 0.721 and a Brier score of 0.161, the model showed good apparent calibration performance in the training set (Figure 6). The Hosmer–Lemeshow test yielded a *p* value of 0.723, indicating good calibration (*p* > 0.05).

#### 3.2.6. Bootstrap Internal Validation and Model Stability Evaluation

Bootstrap resampling with 1000 iterations was performed for internal validation and optimism correction. The apparent AUC was 0.714, with a mean bootstrap training AUC of 0.723 and a mean bootstrap test AUC of 0.707, yielding a mean optimism of 0.016. After optimism correction, the model’s AUC was 0.698 (95% CI: 0.669–0.716) (Table 5). This suggested that the reduction in AUC before and after model correction was minimal, indicating limited evidence of overfitting. Overall, the discriminative ability was moderate.

**Figure 7:**
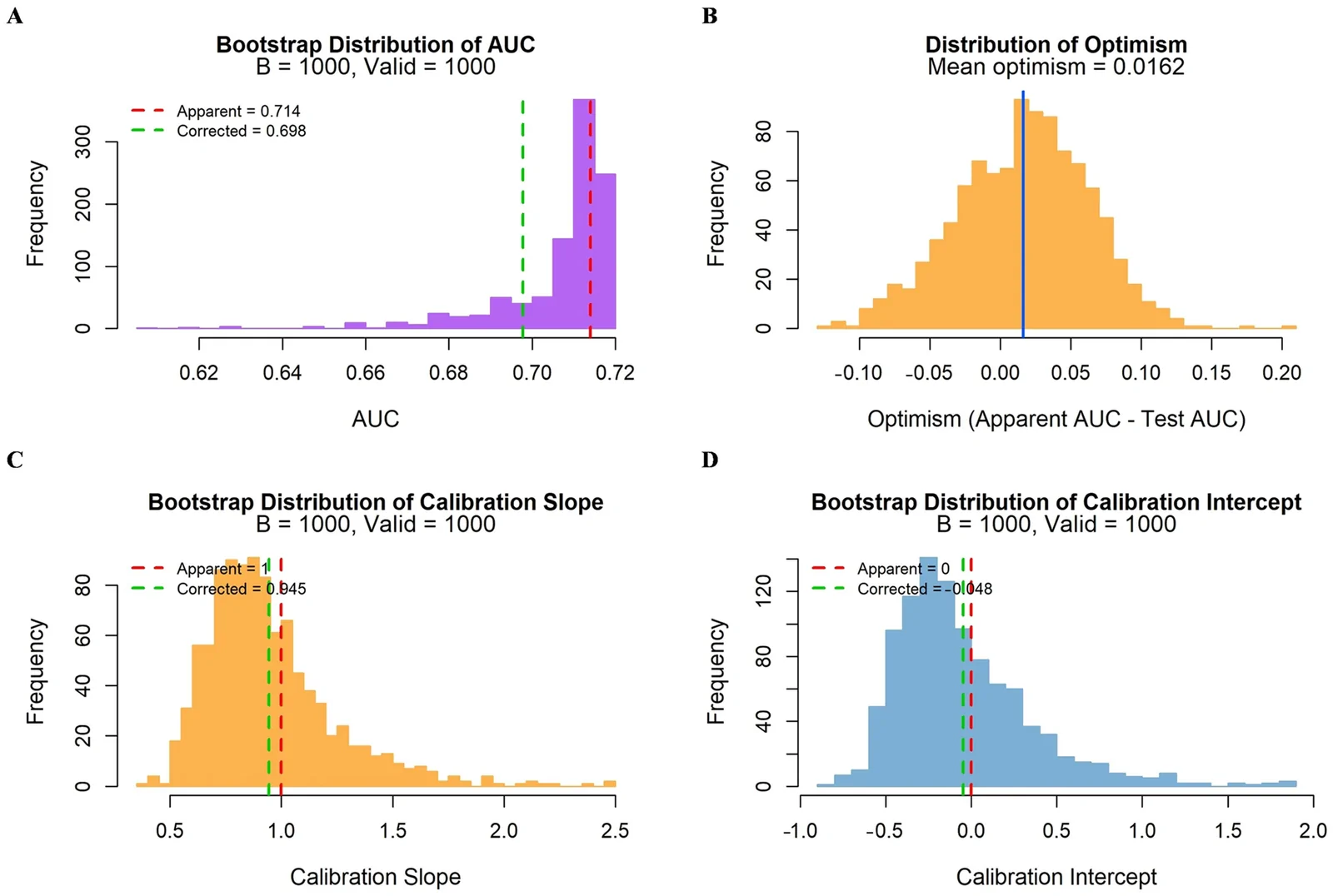
The bootstrap-related parameters of the prediction model. Panel (**A**) illustrates the bootstrap distribution of the AUC, while Panel (**B**) shows the distribution of optimism. Panel (**C**) displays the bootstrap distribution of the calibration slope, and Panel (**D**) depicts the bootstrap distribution of the calibration intercept.

Calibration parameters were analyzed after Bootstrap correction. The apparent calibration intercept was 0, and the optimism-corrected intercept was −0.048 (95% CI: −0.586 to 1.000). The apparent calibration slope was 1, and the corrected slope was 0.945 (95% CI: 0.552 to 1.664) (Table 6). These findings indicate that the calibration intercept remained near zero and the calibration slope remained near 1 after correction, suggesting generally stable calibration after internal validation, although the relatively wide confidence intervals indicated uncertainty in the calibration estimates. The Bootstrap distributions of the AUC, optimism, calibration slope, and calibration intercept are shown in Figure 7.

#### 3.2.7. Decision Curve Analysis

**Figure 8:**
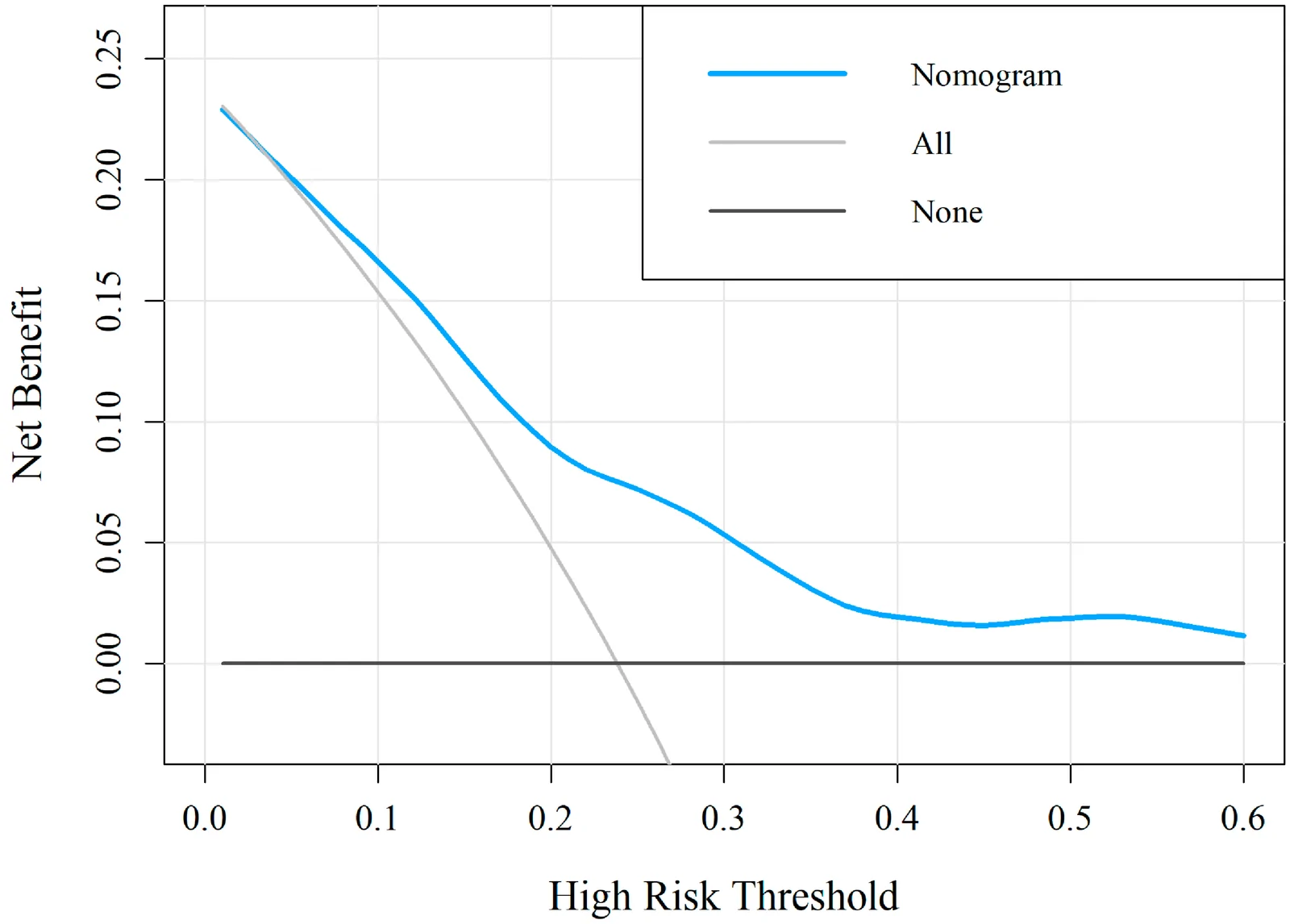
Decision curve analysis of prediction model in training set.

DCA for the training set showed that the model provided a net benefit over both “biopsy-all” and “biopsy-none” strategies across a wide range of threshold probabilities (approximately 0.01–0.48). At selected clinically relevant thresholds, including the risk-stratification boundaries of 0.20 and 0.40 and the Youden index-derived cutoff of 0.30, the model achieved net benefits of 0.082, 0.049, and 0.016 at threshold probabilities of 0.20, 0.30, and 0.40, respectively. At a threshold probability of 0.30, the model yielded a net benefit of 0.049, compared with −0.088 for the “biopsy-all” strategy and 0 for the “biopsy-none” strategy. These findings suggest potential decision-analytic value of the model at the selected clinically relevant thresholds (Figure 8). However, external validation is required to confirm the generalizability of these findings. In the internal validation set, DCA showed limited net benefit, likely due to the small sample size (*n* = 63), further supporting the need for external validation in larger cohorts (Supplementary Figure S2).

### 3.3. Patient Risk Stratification

The low-risk group consisted of 75 cases (51.0% of the population), with 11 csPCa cases detected (14.7%). The intermediate-risk group comprised 53 cases (36.1%), with 15 csPCa cases (detection rate: 28.3%). The high-risk group comprised 19 cases (12.9%), with 9 csPCa cases (detection rate: 47.4%) (Table 7). The differences in detection rates among the three groups were statistically significant (χ^2^ = 9.859, *p* = 0.007), with a significantly higher detection rate in the high-risk group compared with the low-risk group (*p* = 0.0018).

## 4. Discussion

### 4.1. Main Research Findings

csPCa risk prediction for patients with PSA gray zone (4–10 ng/mL) has been shown to be difficult [15]. PSA levels alone are insufficient to reliably distinguish csPCa from benign prostate conditions, which may lead to unnecessary biopsies or missed diagnoses [2,16]. In this study, we developed a risk prediction model incorporating clinical and MRI quantitative parameters for this population, and evaluated its discriminatory ability, calibration, internal stability, and potential decision-analytic value [17,18].

Our results showed that age, ADC value and relative T2 value were associated with csPCa in univariate analysis. Multivariate logistic regression analyses identified age and relative T2 value as independent predictors of csPCa, while PSA and ADC were not included in the primary model. The AUCs for the primary prediction model were 0.720 in the training set and 0.711 in the internal validation set. Bootstrap internal validation yielded an optimism-corrected AUC of 0.698, with a mean optimism of 0.016, suggesting limited evidence of overfitting. Overall, these findings suggest that the model may provide supplementary information for csPCa risk assessment in patients within the PSA gray zone, although its moderate discrimination and the uncertainty of some parameter estimates warrant cautious interpretation.

We also performed exploratory risk stratification based on the predicted probabilities. The csPCa detection rates were 14.7% in the low-risk group, 28.3% in the intermediate-risk group, and 47.4% in the high-risk group, showing a stepwise increase with ascending risk level. These findings suggest that the model-derived probabilities may help differentiate patients with different estimated risks of csPCa within the PSA gray zone. However, the proposed risk categories and probability thresholds were exploratory and have not been externally validated as clinical decision thresholds. Therefore, the model should be considered a supplementary risk-assessment tool and interpreted in conjunction with other clinical and imaging findings rather than used as a standalone basis for biopsy decisions.

### 4.2. Predictive Value of Age and Relative T2 Value

Age is a well-established risk factor for PCa and has also been associated with an increased risk of csPCa. With advancing age, prostate epithelial cells are increasingly susceptible to chronic inflammation, hormonal changes, and cumulative genetic damage, which collectively contribute to an elevated risk of csPCa. In our multivariate logistic regression analysis, age was identified as a statistically significant independent predictor of csPCa risk in the PSA gray zone (OR = 1.11, 95% CI: 1.02–1.20, *p* = 0.017), suggesting that increasing age was associated with a higher probability of csPCa in this cohort.

The relative T2 value was a key predictor in the primary model [9]. Unlike the direct measurement of the T2 signal intensity, the relative T2 value was calculated by normalizing the signal intensity of the lesion area to that of normal-appearing prostate tissue at the same level, which may reduce the effects of scanning equipment, acquisition settings, and individual differences on signal intensity [19,20]. In our multivariate analysis, a higher relative T2 value was associated with an increased probability of csPCa (OR = 22.40, 95% CI: 1.88–267.19, *p* = 0.014). However, the wide confidence interval indicates substantial uncertainty and limited precision in the estimated effect size, which may be related to the limited number of csPCa events and the relatively small sample size. Therefore, although relative T2 value showed potential as a quantitative imaging predictor of csPCa in this cohort, the magnitude and stability of its association should be interpreted cautiously and require further validation in larger independent cohorts. The ADC value was statistically significant in the univariate analysis, but not an independent predictor in the multivariate analysis. This finding may reflect the limited sample size, overlapping information between quantitative MRI parameters, or overlapping ADC characteristics between csPCa and benign lesions in this specific PSA gray-zone population. Given the established biological and imaging relevance of ADC, its exclusion from the primary model should not be interpreted as evidence of no predictive value. Therefore, ADC was additionally forced into the model in a sensitivity analysis to assess whether it provided incremental predictive value beyond age and relative T2 value. Forcing ADC into the model did not materially improve model performance (AUC 0.721 vs. 0.720 for the primary model), suggesting limited incremental predictive value in this cohort.

### 4.3. Model Diagnostic Power and Stability

The prediction model combining age and relative T2 value achieved an AUC of 0.720 in the training set and 0.711 in the internal validation set, indicating moderate discriminative ability. The similar AUC values between the training and internal validation sets suggest that the model showed relatively stable discrimination within this cohort, with limited evidence of substantial overfitting. However, the overall discriminatory performance remained moderate, and the model should not be used as a standalone test for csPCa diagnosis or as the sole basis for biopsy decision-making. External validation in independent multicenter cohorts is required before broader clinical application.

Bootstrap internal validation supported the model’s stability and moderate discriminative performance [21]. After 1000 Bootstrap resampling iterations with optimism correction, the AUC decreased from 0.714 to 0.698, suggesting limited evidence of overfitting. After correction, the calibration slope approached 1, and the calibration intercept approached 0, suggesting generally stable calibration, although the relatively wide confidence intervals indicated uncertainty in these estimates. The corrected AUC of approximately 0.70 further indicates that the overall discriminative performance remains moderate and may potentially be improved by incorporating additional clinical and quantitative imaging variables. The likelihood ratio test showed improved model fit after adding relative T2 value to age; however, given the uncertainty observed in other incremental performance measures, the additional predictive contribution of relative T2 value should be interpreted cautiously and confirmed in larger independent cohorts.

Calibration analysis showed agreement between the predicted and observed probabilities in the training set. However, given the limited sample size, the calibration results should be interpreted cautiously. Further assessment of calibration performance in larger independent and multicenter cohorts is warranted to evaluate the reproducibility of the model across different patient populations and clinical settings [22,23].

### 4.4. Risk Stratification and Potential Decision-Analytic Value

Risk stratification based on predicted probabilities provided additional clinically relevant information beyond reporting the model AUC alone [24]. Patients were classified into the low-, intermediate-, and high-risk groups according to the predefined probability thresholds. The csPCa detection rate increased progressively across the three risk categories, from 14.7% in the low-risk group to 28.3% in the intermediate-risk group and 47.4% in the high-risk group. These findings suggest that the model-derived probabilities may help differentiate patients with varying levels of csPCa risk within the PSA gray zone. However, these risk categories and probability thresholds were exploratory and have not been externally validated as clinical action thresholds. Therefore, the proposed stratification should be interpreted as a supplementary risk-assessment framework rather than a standalone basis for biopsy decision-making and should be considered together with other clinical and imaging findings [25]. DCA in the training set showed a higher net benefit than the “biopsy-all” and “biopsy-none” strategies at selected clinically relevant threshold probabilities, suggesting potential decision-analytic value [26]. Nevertheless, given the single-center retrospective design, the limited number of csPCa events, and lack of external validation, the clinical applicability of the model remains uncertain. Further prospective multicenter validation and evaluation of additional clinical and quantitative MRI parameters are required.

### 4.5. Relationship with Previous Studies

Previous studies have shown that the PCa detection rate in the PSA gray zone is relatively low, and that PSA alone is insufficient to guide biopsy decisions. Developing predictive models with clinical and MRI quantitative features has also been proposed to improve diagnostic accuracy in the PSA gray zone [27,28]. Unlike approaches relying on a single PSA indicator, we combined age and relative T2 value to assess csPCa risk [29]. We used the relative T2 value as a standardized MRI parameter for risk assessment in patients with PSA levels in the 4–10 ng/mL gray zone. Compared with absolute T2 signal intensity, the relative T2 value may be more reproducible and facilitates quantitative comparisons across patients. We also visualized the model as a nomogram.

We considered incorporating other clinically relevant variables, including PI-RADS score, PSA density, free-to-total PSA ratio, and prostate volume, but these were not included in the primary model for three reasons: ① PI-RADS scoring is subjective and may compromise reproducibility; ② PSA density and free-to-total PSA ratio were not consistently available in this retrospective cohort, and including them would have reduced the effective sample size; ③ our goal was to develop a parsimonious model based on routinely available, objective indicators. Therefore, we cannot conclude that our model outperforms PI-RADS or other established diagnostic strategies. Accordingly, our model was intended as a complementary risk-assessment tool when these standard variables are unavailable or incompletely recorded, rather than as a replacement for PI-RADS- or PSA density-based strategies. Future studies should incorporate these variables to clarify their incremental diagnostic value.

### 4.6. Limitations

This study has several limitations. First, this is a single-center retrospective study with a relatively small sample size, and both the training and internal validation sets were derived from the same center, which limits the external validity of our findings. Additionally, the wide confidence interval for the odds ratio of relative T2 value reflects the limited sample size and the relatively small number of csPCa events, which limits the precision of this estimate. Therefore, independent external validation in multicenter cohorts is required before broader clinical application [30]. Second, we did not stratify patients according to Gleason score or ISUP grade beyond the csPCa threshold, nor did we differentiate between different grade groups within csPCa. Therefore, our model predicts overall csPCa risk rather than distinguishing between different levels of disease aggressiveness. This limitation may restrict the model’s ability to provide more refined risk stratification among patients with csPCa. Third, key clinical variables such as prostate volume, PSA density, free-to-total PSA ratio, digital rectal examination results, and PI-RADS score were not included, which may have reduced the predictive performance of the model. We acknowledge that the exclusion of PI-RADS and other established variables limits direct comparison with existing diagnostic algorithms. However, our intention was not to replace PI-RADS-based strategies but to provide a complementary, objective risk-assessment tool based on routinely available quantitative indicators. Fourth, pathological confirmation was based on either prostate biopsy or radical prostatectomy specimens. Although radical prostatectomy specimens were used as the reference standard when both were available, differences between biopsy- and prostatectomy-based pathological assessment may have introduced reference-standard heterogeneity. In addition, biopsy technique was not incorporated into the prediction model, which may have influenced pathological ascertainment. Fifth, tumor location within the prostate, including peripheral-zone and transition/central-zone lesions, was not incorporated into the current analysis. Given the known differences in MRI performance and imaging characteristics across prostate zones, lesion location may influence the predictive value of quantitative MRI parameters and should be evaluated in future studies. Sixth, although the model demonstrated good calibration in the training set (Hosmer–Lemeshow *p* = 0.723), calibration performance was less favorable in the internal validation set (Hosmer–Lemeshow *p* < 0.001). This discrepancy may reflect the limited sample size of the validation cohort (*n* = 63, with only 15 csPCa events) and suggests that the model’s calibration performance may be unstable in small datasets. This further underscores the necessity of external validation in larger cohorts before any clinical application. Seventh, although bootstrap correction was applied for internal validation, the optimism-corrected AUC remained approximately 0.70, indicating moderate discriminative performance. While logistic regression was selected for its interpretability and suitability for the present sample size, alternative modeling approaches may be explored in larger cohorts. Finally, the proposed risk-stratification thresholds were exploratory and have not been externally validated as clinical decision thresholds; their stability and clinical applicability should therefore be evaluated in prospective multicenter cohorts [31,32,33].

### 4.7. Research Prospects

Future research could be advanced in several key areas. First, a multicenter, large-sample prospective study should externally validate this model and evaluate its stability across different institutions, MRI platforms, and patient populations. Second, factors such as PSA density, free-to-total PSA ratio, prostate volume, PI-RADS score, and other quantitative MRI parameters could be evaluated to determine whether their incorporation improves predictive performance. Third, future studies should incorporate tumor location (peripheral zone vs. transition/central zone) as a potential predictor to evaluate whether lesion location provides incremental predictive value beyond age and relative T2 value, and should further stratify csPCa according to Gleason score or ISUP Grade Group to assess whether the model can distinguish different levels of disease aggressiveness. Fourth, after adequate external validation, the model could be developed into a clinical calculator or embedded into decision-support tools to facilitate individualized risk assessment in clinical practice.

## 5. Conclusions

We constructed a binary classification model for predicting csPCa risk using age and relative T2 value as predictors. The model was visualized as a nomogram and demonstrated moderate discriminative performance in both the training and internal validation sets. Bootstrap internal validation showed limited evidence of overfitting and generally stable calibration. Risk stratification based on predicted probabilities demonstrated progressively increasing csPCa detection rates across the low-, intermediate-, and high-risk groups.

Overall, the model may provide supplementary information for individualized csPCa risk assessment in patients within the PSA gray zone. However, its discriminative performance remains moderate, and the proposed risk-stratification thresholds should not be used as standalone clinical decision thresholds. The model should be interpreted together with other clinical and imaging findings. Further prospective, multicenter external validation in larger and more diverse populations is required before broader clinical application.

## Figures and Tables

**Table 1:** Comparison of baseline characteristics between csPCa and non-csPCa groups.

Parameter	csPCa Groups (*n* = 50)	Non-csPCa Groups (*n* = 160)	Statistical Values (Z/t)	*p*-Value
Age (years)	65.5 (62.0–70.0)	64.0 (61.0–67.0)	Z = −2.116	0.0344
PSA (ng/mL)	6.75 (6.13–7.48)	6.73 (6.07–7.41)	Z = −0.361	0.7179
ADC (×10^−3^ mm^2^/s)	1.09 ± 0.15	1.17 ± 0.17	t = −3.468	0.0006
Relative T2 value	1.20 ± 0.15	1.10 ± 0.17	t = −3.789	0.0002

**Table 2:** Interobserver agreement test (ICC).

Parameter	ICC Estimate	95% Confidence Interval	Reliability Interpretation
ADC value			
Single-measure ICC	0.921	0.897–0.939	Excellent
Average-measure ICC (2 observers)	0.959	0.946–0.969	Excellent
Relative T2 value			
Single-measure ICC	0.930	0.910–0.947	Excellent
Average-measure ICC (2 observers)	0.964	0.953–0.973	Excellent

ICC > 0.90 = excellent; 0.75–0.90 = good; 0.5–0.75 = moderate; <0.50 = poor.

**Table 3:** Comparison of baseline characteristics between training and internal validation sets.

Parameter	Training Set (*n* = 147)	Internal Validation Set (*n* = 63)	Statistical Values (Z/t/χ^2^)	*p*-Value
Age (years)	64.0 (62.0–67.0)	64.0 (61.3–68.0)	Z = −0.147	0.4450
PSA (ng/mL)	6.73 (6.01–7.42)	6.76 (6.18–7.46)	Z = −0.211	0.8332
ADC (×10^−3^ mm^2^/s)	1.10 ± 0.15	1.12 ± 0.17	t = 0.905	0.3663
Relative T2 value	1.12 ± 0.17	1.13 ± 0.18	t = 0.423	0.6730
Pathological type (csPCa/non-csPCa, *n* (%))	35 (23.8%)/112 (76.2%)	15 (23.8%)/48 (76.2%)	χ^2^ = 0	1.000

**Table 4:** Multivariate logistic regression analysis of factors associated with pathological outcome.

Variable	Partial β	SE	Wald χ^2^	*p*-Value	OR (95% CI)
Age (year)	0.10	0.04	5.65	0.0174	1.11 (1.02–1.20)
ADC (×10^−3^ mm^2^/s)	2.15	1.44	2.23	0.1354	8.62 (0.51–145.92)
Relative T2 value	3.11	1.26	6.04	0.0140	22.40 (1.88–267.19)
Constant	−13.75	3.49	15.56	0.0001	—

**Table 5:** AUC performance of the prediction model based on bootstrap internal validation.

Metric	Apparent (P_App)	Mean P_Boot	Mea P_Test	Meanoptimism (P_Boot − P_Test)	Optimism-Corrected (P_App − O)	95% CI (Percentile)
AUC	0.714	0.723	0.707	0.016	0.698	(0.669, 0.716)

**Table 6:** Calibration metrics of the prediction model based on bootstrap internal validation (B = 1000).

Calibration Metric	Apparent (Estimate)	Mean_Boot	Mean_Test	Mean Optimism	Optimism Corrected	95% CI (Percentile)
Intercept	0	0	−0.048	0.048	−0.048	(−0.586, 1)
Slope	1	1	0.945	0.055	0.945	(0.552, 1.664)

**Table 7:** csPCa detection rate among different model-based risk groups.

Risk Group	Predicted Probability Range	*n*	Proportion	Proportion (%)	csPCa Cases	Non-csPCa Cases	csPCa Detection Rate
Low-risk	*p* < 0.20	75	75/147	51.0%	11	64	14.7%
Intermediate-risk	0.20 ≤ *p* < 0.40	53	53/147	36.1%	15	38	28.3%
High-risk	*p* ≥ 0.40	19	19/147	12.9%	9	10	47.4%

## Data Availability

The datasets generated and analyzed during the current study are available from the corresponding author on reasonable request.

## Supplementary Materials

The following supporting information can be downloaded at: https://www.mdpi.com/article/10.3390/diagnostics16172771/s1, Supplementary Figure S1: Calibration curve of the prediction model in the validation set; Supplementary Figure S2: Decision curve analysis of prediction model in the validation set; Supplementary Figure S3: ROC curve of the extended model after forcing ADC into the final model (age + relative T2 value + ADC) in the training set.
