# Supplementary material for: Stratified Analysis of Patients Within the PSA Gray Zone (4–10 ng/mL) and Its Clinical Application Value: Development of a Predictive Model for Clinically Significant Prostate Cancer Using Quantitative Indicators of PSA, ADC, and Relative T2 Value
Source: Diagnostics (Basel). 2026 Aug 28;16(17):2771. doi: 10.3390/diagnostics16172771 (PMC13564744; doi:10.3390/diagnostics16172771)
Supplement: Supplementary file 1 [file diagnostics-16-02771-s001.zip › diagnostics-4504086-supplementary.pdf]

Supplementary Figure S1. Calibration curve of the prediction model in the validation set.

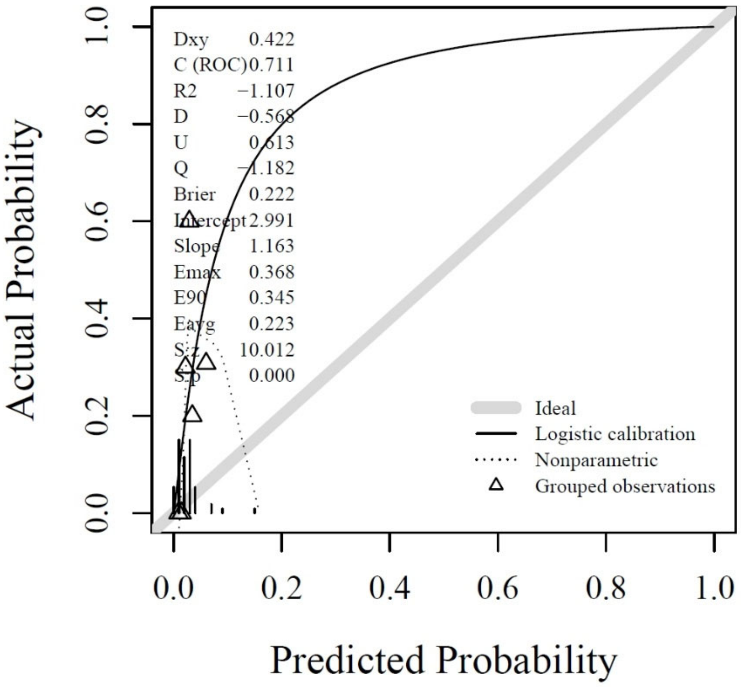

The calibration curve shows the relationship between predicted and observed probabilities. The Hosmer-Lemeshow test yielded a P value < 0.001, and the Brier score was 0.222, indicating less favorable calibration in this small validation cohort (n=63).

Supplementary Figure S2. Decision curve analysis of prediction model in the validation set.

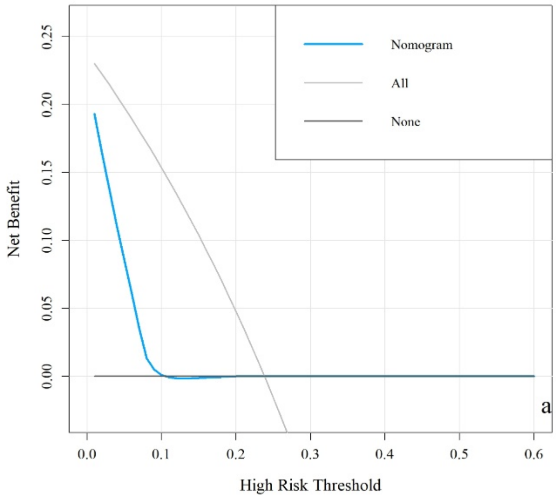

The x-axis represents the threshold probability, and the y-axis represents the net benefit. The model showed limited net benefit, likely due to the small sample size (n=63) and the limited number of csPCa events (n=15), further supporting the need for external validation in larger cohorts.

Supplementary Figure S3. ROC curve of the extended model after forcing ADC into the final model (age + relative T2 value + ADC) in the training set.

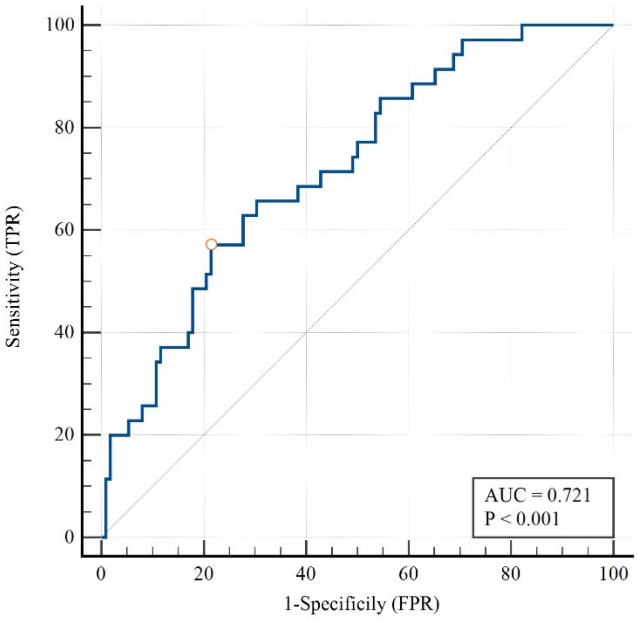

The AUC was 0.721 (P < 0.001), similar to the primary model (AUC = 0.720), indicating that ADC did not provide incremental predictive value beyond age and relative T2 value.
